# Coronary Artery Disease Prevalence in an Executive Population at a Tertiary Medical Center: Protocol for a Retrospective Cohort Study

**DOI:** 10.2196/72451

**Published:** 2025-09-17

**Authors:** Murali Duggirala, Basant Katamesh, Ann Vincent, Ivana Croghan, Brian Dougan, Jayanth Adusumalli, Ryan Hurt, Sanjeev Nanda, Donna Lawson, Amirala Pasha

**Affiliations:** 1 Division of General Internal Medicine Mayo Clinic Rochester, MN United States; 2 Division of Hospital Internal Medicine Mayo Clinic Rochester, MN United States

**Keywords:** coronary heart disease, executive patients, prevalence, risk factors, age stratification, gender stratification, cardiovascular risk

## Abstract

**Background:**

Coronary artery disease (CAD) is a leading cause of global morbidity and mortality. Although CAD prevalence in the general population is well-documented, its occurrence among executive patients remains largely unexplored. An executive is an individual in a major leadership role, such as a C-suite officer, senior manager, board member, trustee, founder, or business owner, responsible for high-level decision-making and strategic direction. These roles often involve demanding schedules and significant stress. Despite their influence and better access to health care, this demographic faces unique challenges such as demanding work schedules, chronic stress, frequent travel, and reduced control over lifestyle. To address executives’ unique health needs, many health care organizations offer specialized programs emphasizing preventive cardiovascular care, using advanced tools such as lipid panels, stress tests, and coronary calcium scans not typically included in primary care, to detect risks early and to promote long-term wellness.

**Objective:**

This protocol aims to design a study to determine the prevalence of CAD in executive patients and compare it to the established prevalence in the US general population with the overarching goal of improving screening and care of CAD among executive patients.

**Methods:**

This protocol proposes a retrospective review of medical records for patients with CAD seen at the Mayo Clinic’s Executive Health Program from January 1, 2020, to December 31, 2023, with the aim of determining the prevalence of CAD in executive patients. The primary outcome is CAD prevalence, which will be identified through clinical diagnoses in the electronic medical records. Secondary outcomes include demographics, cardiovascular medications, social determinants of health, laboratory and diagnostic results, coronary calcium scores, and treatment interventions. The prevalence of CAD will be calculated as the proportion of patients with a documented CAD diagnosis relative to the total number of patients in the study cohort.

**Results:**

A total of 24,272 patients were seen in the executive health clinic between January 1, 2020, and December 31, 2023. After applying the inclusion criteria, 6466 executive patients were eligible, with 3290 identified as having a potential CAD diagnosis pending confirmation through a detailed chart review.

**Conclusions:**

In this protocol, we outline a research design and methodology to address a critical gap in understanding the prevalence of CAD among executive patients. This demographic is often overlooked despite their unique risk factors such as high stress and lifestyle choices.

**International Registered Report Identifier (IRRID):**

DERR1-10.2196/72451

## Introduction

Coronary artery disease (CAD) is a major global health issue, significantly contributing to both illness and death [1,2]. In the United States, heart disease is the leading cause of death, with CAD being the primary contributor [3]. CAD significantly impacts overall health care expenditure and incurs additional costs beyond direct medical expenses, such as lost productivity from premature death and disability [4,5]. Key risk factors for CAD include hypertension, diabetes, dyslipidemia, smoking, and physical inactivity [6,7]. These factors accelerate atherosclerosis, a complex condition involving endothelial dysfunction, abnormal lipid metabolism, and inflammatory mechanisms, leading to the accumulation of low-density lipoprotein and other inflammatory particles that form fatty streaks and plaques prone to rupture [6-8]. There are notable disparities in CAD prevalence across different demographics. For example, CAD prevalence increases with age, with only 1% of adults aged 18-44 years reporting CAD, compared to 24.2% of adults aged 75 years and older [9]. Gender differences are also significant, with men reporting higher rates of CAD than women [10]. From 2009 to 2019, 7% of men reported CAD in 2019 compared to 4.2% of women [10]. Black and Hispanic populations face greater risks, partly due to higher rates of hypertension, diabetes, and obesity [11].

Socioeconomic factors also play a critical role; individuals from lower socioeconomic backgrounds often have limited access to health care, nutritious food, and opportunities for physical activity, increasing their risk for CAD [12,13]. Although insurance coverage is not reported to influence CAD prevalence, factors such as education level and income are reported to have a significant impact [14].

In recent decades, many health care organizations have launched executive health programs tailored specifically for the medical care of professionals in leadership roles [15-17]. Unlike traditional primary care, these programs place a stronger emphasis on preventive care, particularly cardiovascular screening with advanced lipid testing, cardiac stress testing, and coronary artery calcium scan, which are not routinely implemented in primary care [15-17]. Some programs also offer access to emerging disease screening technologies, including predictive genetic testing, pharmacogenomic screening, blood-based multi-cancer screening, and whole-body magnetic resonance imaging [16]. The overarching goal of these programs is to identify health risks and diseases early, promote overall wellness, and provide efficient, accessible health care with a strong focus on disease prevention [15-17].

The target demographic for these executive health programs are typically individuals who hold major leadership positions in corporations, businesses, and trusts, including business owners and entrepreneurs, who often face demanding and stressful professional schedules, such as C-suite executives, senior management, board members, trustees, founders, and business owners, all of whom collectively drive economic activity and contribute significantly to the overall health of the economy [15-17]. Although executives generally have better access to health care, higher incomes, and higher educational levels compared to the general population, they also face high-stress work environments that often interfere with their ability to maintain healthy lifestyle habits [15-19]. Additionally, there are demographic variations between this population and the general population. For instance, in terms of race and ethnicity, executive health program participants often reflect a more homogeneous group, frequently from higher socioeconomic backgrounds, which can influence health outcomes [20]. Gender disparities are notable as well, with a predominance of male participants, aligning with the typical gender distribution in executive roles [20]. The age group of these individuals typically ranges from 40 to 60 years—a period during which the risk for CAD significantly increases [14].

This combination of factors underscores the need to investigate CAD prevalence among executives to develop targeted interventions tailored to their unique needs. However, there are currently no published data on these factors or the prevalence of CAD among executives, representing a critical gap in the literature. We aim to conduct a retrospective cohort study to determine the prevalence of CAD among individuals enrolled in an executive health program at a large medical center.

## Methods

### Study Design

The design of this study is a retrospective chart review of executives who received care at The Executive Health Program at Mayo Clinic in Rochester, Minnesota, from January 1, 2020, to December 31, 2023. This study protocol was developed in accordance with STROBE (Strengthening the Reporting of Observational Studies in Epidemiology) guidelines for observational research (Multimedia Appendix 1) [21,22].

### Recruitment

#### Participants

The population for this proposed study includes executive patients who were enrolled in the Executive Health Program at the Mayo Clinic in Rochester site between January 1, 2020, and December 31, 2023. We define executive patients as individuals holding major leadership positions in corporations, businesses, and trusts, including business owners and entrepreneurs. This group encompasses C-suite executives, senior management, board members, trustees, founders, and business owners who collectively drive economic activity and significantly contribute to the overall health of the economy.

#### Inclusion Criteria

**Figure 1:**
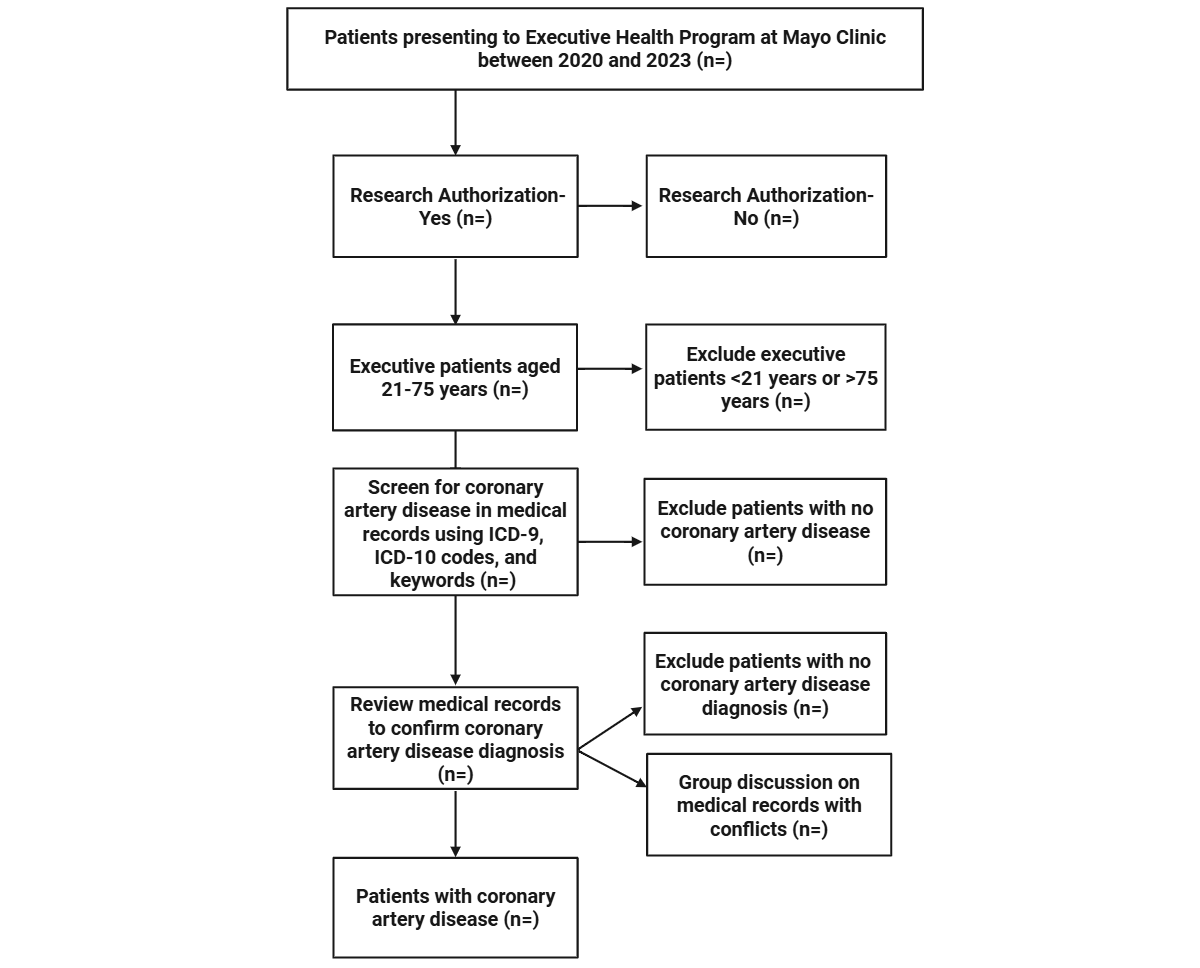
Complete screening and inclusion process. ICD-9: International Classification of Diseases, Ninth Revision; ICD-10: International Classification of Diseases, Tenth Revision.

This protocol proposes to include patients aged 21 to 75 years who sought care at the Executive Health Program between January 1, 2020, and December 31, 2023; provided prior research authorization; and have a CAD diagnosis. We propose screening the medical records of the patient cohort who presented during this period by using the codes of the International Classification of Diseases, Ninth Revision; International Classification of Diseases, Ninth Revision, Clinical Modification; International Classification of Diseases, Tenth Revision; and International Classification of Diseases, Tenth Revision, Clinical Modification, as described in Table 1, in addition to keywords such as coronary artery, coronary artery disease, and atherosclerosis. We will utilize an institutionally developed, validated, and research-approved electronic search tool, Mayo Data Explorer, for the screening. Medical records that pass the screening process will be reviewed in detail to confirm CAD diagnosis within a clinical note and/or documentation of CAD through a radiology/cardiology clinical test. Conflicts that arise regarding medical records with an unclear diagnosis will be resolved through group discussions. The details of the complete screening and inclusion are outlined in Figure 1.

**Table 1:** ICD^a^ codes used for patient prescreening.

ICD codes		Description
**ICD-9^b^** **and ICD-9-CM^c^** **code**		
	414.00	Coronary atherosclerosis of unspecified type of vessel, native or graft
	414.01	Coronary atherosclerosis of native coronary artery
**ICD-10^d^** **and ICD-10-CM^e^** **code**		
	I25.10	Atherosclerotic heart disease of native coronary artery without angina pectoris
	I25.110	Atherosclerotic heart disease of native coronary artery with unstable angina pectoris
	I25.111	Atherosclerotic heart disease of native coronary artery with angina pectoris with documented spasm
	I25.118	Atherosclerotic heart disease of native coronary artery with other forms of angina pectoris
	I25.119	Atherosclerotic heart disease of native coronary artery with unspecified angina pectoris

^a^ICD: International Classification of Diseases.

^b^ICD-9: International Classification of Diseases, Ninth Revision.

^c^ICD-9-CM: International Classification of Diseases, Ninth Revision, Clinical Modification.

^d^ICD-10: International Classification of Diseases, Tenth Revision.

^e^ICD-10-CM: International Classification of Diseases, Tenth Revision, Clinical Modification.

#### Sample Size Calculation

Study entry criteria will include being an empaneled patient in the Mayo Clinic Executive Health Program, having provided authorization for research, and aged 21 to 75 years. Based on clinical experience, we anticipate that approximately 5%-10% of the executive patients seen during that time period will meet the inclusion criteria. With as estimated denominator of 25,000 and an expected CAD prevalence of 5%-10%, the margin of error for a 95% CI is expected to be less than SD 0.5%. This is based on using nQuery Advisor 7.0 (confidence interval for a proportion using the normal approximation).

### Record Review and Data Collection

#### Primary Outcome Variables to be Abstracted

The primary outcomes of the prevalence of CAD will be determined by a review of medical records of executive patients who presented within this time frame to identify patients with a CAD diagnosis within the clinical notes. Data will be collected and categorized as either binary or continuous into a Research Electronic Data Capture (REDCap) database, as detailed in Table 2.

**Table 2:** Primary outcome variables to be abstracted.

Key outcomes variables	Source	Variable type
Record ID	Chart review	Identifier
Were they diagnosed with coronary artery disease?	Chart review	Binary
Age at coronary artery disease diagnosis	Chart review	Continuous

#### Secondary Outcome Variables to be Abstracted

The secondary outcome variables to be abstracted will include baseline demographic characteristics, cardiovascular-related medications, responses to the Social Determinants of Health Questionnaire (covering exercise, nutrition, and alcohol habits), laboratory test results, diagnostic tests, coronary calcium scan results, and treatment interventions (Tables 3-8). Data will be collected and categorized as binary, continuous, or categorical into a REDCap database as detailed in Tables 3-8.

**Table 3:** Secondary outcomes: baseline demographic variables to be abstracted.

Variable		Source	Variable type
Gender		Chart review	Categorical
Race		Chart review	Categorical
Ethnicity		Chart review	Categorical
Level of education		Chart review	Categorical
Marital status		Chart review	Categorical
**Medical history**		Chart review	Binary, categorical
	Hypertension		
	Chronic kidney disease		
	Hyperlipidemia		
	Diabetes		
	Stroke		
	Obstructive sleep apnea		
	Peripheral vascular disease		
	Heart failure		
**Family history**		Chart review	Binary, categorical
	Coronary artery disease		
	Hypertension		
	Chronic kidney disease		
	Hyperlipidemia		
	Diabetes		
	Stroke		
	Obstructive sleep apnea		
	Peripheral vascular disease		
	Heart failure		
**Physical measurements**			
	Height (cm)	Chart review	Continuous
	Weight (kg)	Chart review	Continuous
	BMI	Chart review	Continuous

**Table 4:** Secondary outcomes: medication use at the time of coronary artery disease diagnosis to be abstracted.

Medication use	Source	Variable type
Are they taking lipid-lowering agents? If yes, which ones?	Chart review: medications	Binary, categorical
Are they taking angiotensin-converting enzyme inhibitors? If yes, which ones?	Chart review: medications	Binary, categorical
Are they taking angiotensin II receptor blockers? If yes, which ones?	Chart review: medications	Binary, categorical
Are they taking β-blockers? If yes, which ones?	Chart review: medications	Binary, categorical
Are they taking calcium-channel blockers? If yes, which ones?	Chart review: medications	Binary, categorical
Are they taking diuretics? If yes, which ones?	Chart review: medications	Binary, categorical
Are they taking antiplatelet agents? If yes, which ones?	Chart review: medications	Binary, categorical
Are they taking anticoagulants? If yes, which ones?	Chart review: medications	Binary, categorical
Are they taking vasodilators and cardiac medications? If yes, which ones?	Chart review: medications	Binary, categorical
Are they taking α-blockers? If yes, which ones?	Chart review: medications	Binary, categorical
Are they taking colchicine?	Chart review: medications	Binary
Are they taking supplements? If yes, which ones?	Chart review: medications	Binary, categorical

**Table 5:** Secondary outcomes: social determinants of health to be abstracted.

Variable		Source	Variable type
**Exercise habits**			
	On average, how many days per week do you engage in moderate to strenuous exercise (like a brisk walk)?	Social Determinants of Health Questionnaire	Continuous
	On average, how many minutes do you engage in exercise on this level?	Social Determinants of Health Questionnaire	Continuous
	Type of exercise (cardio, strength training, other)	Social Determinants of Health Questionnaire	Categorical
**Dietary habits**			
	On average, how many servings of fruits and vegetables do you eat each day (serving size equal to 1 tennis ball)?	Social Determinants of Health Questionnaire	Continuous
	Do you use extra virgin olive oil as your main source of fat in your diet?	Social Determinants of Health Questionnaire	Binary
**Alcohol consumption**			
	How often do you have a drink containing alcohol? (times per week)	Social Determinants of Health Questionnaire	Continuous
	How many drinks containing alcohol do you have on a typical day when you are drinking?	Social Determinants of Health Questionnaire	Continuous
	How often do you have 6 or more drinks on one occasion?	Social Determinants of Health Questionnaire	Continuous
**Smoking habits**			
	Smoking (any form of tobacco products, eg, chewing): if yes, which type?	Chart review	Binary, categorical
	Did they do smokeless tobacco such as chew or snuff?	Chart review	Binary
	Total packs per year and current packs per day	Chart review	Continuous
	If former smoker, the date they quit smoking or smokeless tobacco	Chart review	Date
	Number of packs per year they used to smoke	Chart review	Continuous
	e-Cigarette and vaping use	Chart review	Binary

**Table 6:** Secondary outcomes: laboratory test results to be abstracted.

Variable	Source	Variable type
Total cholesterol	Chart review: labs^a^	Continuous
High-density lipoprotein cholesterol	Chart review: labs	Continuous
Low-density lipoprotein cholesterol	Chart review: labs	Continuous
Triglycerides	Chart review: labs	Continuous
Lipoprotein(a)	Chart review: labs	Continuous
Apolipoprotein A1	Chart review: labs	Continuous
Apolipoprotein B	Chart review: labs	Continuous
Fasting glucose	Chart review: labs	Continuous
Hemoglobin A_1c_	Chart review: labs	Continuous
Fibrinogen	Chart review: labs	Continuous
C-reactive protein	Chart review: labs	Continuous
High-sensitivity C-reactive protein	Chart review: labs	Continuous
Myocardial infarction–heart ceramide risk score	Chart review: labs	Continuous

^a^Labs: laboratory test results.

**Table 7:** Secondary outcomes: diagnostic test information variables to be abstracted.

Variable		Source	Variable type
**Diagnostic tests**			
	Did the patient undergo an exercise stress test?	Chart review	Binary
	Did the patient undergo an echocardiogram?	Chart review	Binary
	Did the patient undergo an electrocardiogram?	Chart review	Binary
	Did the patient undergo a nuclear stress test?	Chart review	Binary
	Did the patient undergo a heart computed tomography?	Chart review	Binary
	Did the patient undergo a cardiac catheterization?	Chart review	Binary
**Calcium scan**			
	Was a calcium scan done?	Chart review	Binary
	Date of calcium scan	Chart review	Date
	Total score of calcium	Chart review	Continuous
	Left anterior descending artery measurement	Chart review	Continuous
	Circumflex measurement	Chart review	Continuous
	Left main vessel measurement	Chart review	Continuous
	Right coronary artery measurement	Chart review	Continuous
	Percentile	Chart review	Continuous

**Table 8:** Secondary outcomes: procedures and treatment interventions related to coronary artery disease that have to be abstracted.

Variable	Source	Variable type
Did the patient undergo angioplasty?	Chart review: procedures	Binary
Did the patient receive a stent?	Chart review: procedures	Binary
Did the patient undergo coronary artery bypass grafting?	Chart review: procedures	Binary

### Abstractor Training

Prior to the commencement of data extraction, a comprehensive training reference guide will be developed to delineate the specific objectives of the project. This guide will serve as an essential resource, with any updates or modifications promptly incorporated and communicated to the team. Training sessions will be conducted for each new abstractor to ensure thorough preparation for the extraction process.

### Quality Assurance

To ensure the accuracy and reliability of the extracted data, we will implement random spot checks on the REDCap dashboard. Our goal is to achieve a 10% spot-check rate, equating to approximately 10 checks per page, with each page containing 100 patient records. These checks will be assigned randomly to minimize bias and will be conducted by reviewers independent of the initial data extractors. This dual-review approach is crucial for identifying any discrepancies or errors that may have been initially overlooked. If errors are detected, they will be promptly corrected, and the responsible individuals will receive retraining to address any gaps in their understanding.

### Statistical Analysis Plan

Patients will be initially categorized into those with established CAD and those without. The prevalence of CAD among executive patients will be calculated, along with age- and gender-adjusted incidence rates. The prevalence of CAD will be estimated by calculating the proportion of individuals diagnosed with CAD out of the total sample. Confidence intervals for the prevalence estimates will be calculated using the Wilson score method. All secondary variables collected will be summarized using descriptive statistics. For binary variables, frequencies and percentages will be reported. For continuous variables, means, standard deviations, medians, and interquartile ranges will be calculated. Chi-square tests will be used for categorical variables, and 1-sided *t* tests or analysis of variance will be used for continuous variables. Logistic regression models will be employed to adjust for potential confounders and to identify independent predictors of disease prevalence. Statistical analysis will be conducted using SAS software (version 9.4; SAS Institute Inc) [23].

### Ethical Considerations

Patient or public involvement was not incorporated in the design, conduct, reporting, or dissemination plans of this research. Ethical principles have and will continue to be carefully considered and adhered to, and only patients who previously consented to the use of their data for research purposes will be included in this cohort. All data will be deidentified prior to analysis to ensure patient privacy and confidentiality. All procedures are in accordance with the Declaration of Helsinki, and this study underwent expedited review procedures and was determined to be exempt from institutional review board approval (ID 24-005576; 45 CFR 46.104d, category/subcategory 4[iii]).

## Results

A total of 24,272 patients were identified as having been seen in the Executive Health Clinic at Mayo Clinic in Rochester between January 1, 2020, and December 31, 2023. After confirming research authorization and executive status and excluding individuals younger than 21 years or older than 75 years, 6466 patients met the inclusion criteria. Of these, 3290 patients had a mention of CAD in their medical records. This subset of medical records will undergo a secondary review to confirm the diagnosis of CAD and abstract variables as outlined in the study protocol.

## Discussion

This study seeks to address a significant gap in the literature by evaluating the prevalence of CAD within an executive health population—a group that has been largely understudied despite its unique risk profile. Although CAD prevalence is well-characterized in the general US population, its burden among executives remains unclear [14,24]. Given the distinct demographic, occupational, and lifestyle characteristics of this group, we hypothesize that CAD prevalence may differ meaningfully from that of the broader population. Executives often experience elevated levels of work-related stress, irregular schedules, and limited time for physical activity, all of which are known contributors to cardiovascular risk [19,20]. These factors suggest a potential for higher CAD prevalence in this population. Conversely, patients in executive health programs typically have enhanced access to preventive services than what is offered in routine primary care, including routine cardiovascular screening and personalized education, which may facilitate early detection of subclinical disease or even reduce overall disease burden through proactive risk management [15-17]. As such, the observed prevalence may reflect a complex interplay between increased risk exposure and improved access to care.

Two recent studies have described the prevalence of CAD in the US population by using different methodologies. In the first study, Lee et al [14] evaluated CAD prevalence in the United States from 2011 to 2018 by using data from the Behavioral Risk Factor Surveillance System, where individuals self-reported CAD via telephone surveys. The authors reported an overall CAD prevalence of 5.8%-6.2% in the US population [14]. The prevalence was higher in men compared to women (7.7% vs 4.6%, respectively) and in adults older than 65 years compared to those younger than 45 years (7.4% vs 1.6%, respectively) [14]. Notably, there was a clear discrepancy based on income, with lower CAD prevalence in higher-income individuals compared to those with lower incomes (4.1% vs 8.9%, respectively) [14]. In the second study, Khalid et al [24] used data from the Centers for Disease Control and Prevention’s National Center for Health Statistics to evaluate CAD prevalence trends from 2019 to 2022. They reported an overall CAD prevalence of 4.6%-4.9% in the US population, with higher prevalence in males compared to females (6.4% vs 3.6%, respectively) and in adults older than 75 years compared to those aged 65-74 years, 45-64 years, and 18-44 years (19.7% vs 12.3% vs 4.1% vs 0.5%, respectively) [24]. Both studies [14,24] reported disparities in CAD prevalence among different racial and ethnic groups. Lee et al [14] highlighted American Indian or Alaska Native individuals as having the highest prevalence, while Khalid et al [24] found White adults had the highest prevalence. Both studies relied on self-reported data (telephone and interview surveys) to determine CAD presence, which could result in missed diagnoses among individuals who did not participate in the survey, who were unaware of their diagnosis, or who did not engage in primary and preventative care [14,24]. Compared to these 2 studies, although our proposed study focuses only on executives and not on the general population, our proposed methodology is superior in that it relies on documentation in medical records, reducing the likelihood of missed diagnoses. Additionally, our patient population participates in routine cardiovascular prevention beyond what is typically offered in primary care, further lowering the chance of undiagnosed CAD. This distinguishes our methodology and underscores the significance of our study.

This proposed study is crucial for several reasons. Given the lack of literature on CAD within executives, our study aims to address this significant gap and may be the first to evaluate CAD prevalence within this population. If our findings show a higher prevalence of CAD among executives, it may indicate that our executive health program provides superior screening or that executives face higher CAD risks due to stress and lifestyle factors. Conversely, a lower prevalence might reflect the benefits of enhanced care in executive health programs. Additionally, there are limited data on health conditions specific to executives, and this research could prompt further studies on executive health profiles. Finally, understanding CAD prevalence among executives can help health care providers tailor screening practices and optimize interventions for this group, especially if the prevalence is higher than in the general population.

As with other retrospective cohort studies, our study has several limitations. First, there is the potential for missing or inconsistent data, as medical records may be incomplete or vary in documentation quality. Second, selection bias may be present, as the cohort reflects only those executives who chose to participate in our program and may not be representative of the broader executive population. Additionally, our sample characteristics may differ substantially from those of a community-based medical sample or the US general population. As a result, any observed differences in CAD prevalence may reflect the self-selected nature of our cohort rather than true population-level differences. Third, we recognize that stress may be a predictive variable for CAD. However, data related to stress are not routinely collected in the executive health population and are therefore not readily available in the medical records. This limitation may affect our ability to fully assess the role of psychosocial factors in CAD prevalence. Fourth, the retrospective nature of the study limits our ability to control for the confounding variables, which may influence both the quantitative and qualitative aspects of the data collected. Despite these limitations, our study has several notable strengths. It leverages real-world clinical data collected at a world-class medical institution, offering valuable insights into how CAD is diagnosed and managed in routine executive practice. The inclusion of 3 years of data enhances the robustness of our findings by capturing patients who may not attend annually and increasing the likelihood of identifying CAD diagnoses that may have been missed in a single year. Additionally, our reliance on clinician-documented diagnoses, rather than self-reported data or survey responses, improves the accuracy and clinical relevance of our findings.

This proposed study aims to fill a critical gap in health literature by evaluating CAD prevalence among executives, potentially guiding tailored screening practices and interventions to improve cardiovascular outcomes in this unique population. Our study findings will be submitted to a peer-reviewed journal to ensure rigorous academic dissemination. If the opportunity arises, the results will also be presented at national and international conferences to reach a broader academic and clinical audience.

## Appendix

Multimedia Appendix 1 STROBE reporting checklist.

## Abbreviations

CAD: coronary artery disease
REDCap: Research Electronic Data Capture
STROBE: Strengthening the Reporting of Observational Studies in Epidemiology
